# Pathophysiology of androgen-associated endothelial cell dysfunction in phenotype A polycystic ovarian syndrome revealed by iPSCs modeling

**DOI:** 10.3389/fendo.2026.1682793

**Published:** 2026-02-12

**Authors:** Chia-Eng Wu, Chu-Chun Huang, Yung-Jen Hsiao, Chia-Lang Hsu, Tzu-Hsin Chen, Mei-Jou Chen, Hong-Nerng Ho

**Affiliations:** 1 Department of Obstetrics and Gynecology, National Taiwan University Hospital, College of Medicine, National Taiwan University, Taipei, Taiwan; 2 Department of Medical Research, National Taiwan University Hospital, Taipei, Taiwan; 3 Livia Shangyu Wan Chair Professor of Obstetrics and Gynecology, National Taiwan University, Taipei, Taiwan; 4 Research Center for Cell therapy and Regeneration Medicine, Taipei Medical University, Taipei, Taiwan

**Keywords:** androgen, endothelial dysfunction, iPSC (induced pluripotent stem cell), PCOS (polycystic ovarian syndrome), phenotype A

## Abstract

**Introduction:**

Abundant evidence suggests that women with polycystic ovary syndrome (PCOS) have increased metabolic aberrations and cardiovascular risks and present with signs of endothelial cell (EC) dysfunction. However, whether and how androgen is involved in the pathogenesis of EC dysfunction in PCOS remains unclear.

**Methods:**

In this study, induced pluripotent stem cells (iPSCs) were established from three phenotype A PCOS patients (presenting with oligomenorrhea, hyperandrogenism (HA), and polycystic ovarian morphology (PCOM)) and three control participants. These iPSCs were differentiated into ECs (iPSC-derived ECs) using a chemically defined monoculture protocol. To investigate the direct impact of androgen signaling while excluding confounding effects from aromatization into estrogen, dihydrotestosterone (DHT), a potent, non-aromatizable androgen, was used to treat the iPSC-derived ECs from all subjects (three control and three PCOS iPSC-EC lines). Statistical analyses were performed using t-test and one-way or two-way ANOVA followed by appropriate *post-hoc* tests (p < 0.05).

**Results:**

Single-cell transcriptomic analysis revealed intrinsic differences in cell cycle process, vascular endothelial growth factor (VEGF) signaling, apoptosis, and androgen signaling in PCOS iPSC-derived ECs. Decreased expression of cell proliferation- and cell cycle-related genes was noted in the PCOS iPSC-derived ECs. Functionally, DHT treatment significantly enhanced cell proliferation and angiogenesis in control iPSC-derived ECs in a dose-dependent manner, as demonstrated by increased tube formation and accelerated wound healing. In contrast, these stimulatory effects were blunted in PCOS iPSC-ECs, particularly at physiological concentrations. These functional impairments were associated with the dysregulation of the androgen receptor (AR)/cyclin-dependent kinase 1 (CDK1)/VEGF signaling pathway.

**Discussion:**

In conclusion, disease-specific iPSCs were successfully generated from phenotype A PCOS patients, providing a robust platform for disease modeling in this distinct subgroup. PCOS iPSC-derived ECs exhibited significantly impaired intrinsic and androgen-induced cell proliferation and angiogenesis. These findings offer novel mechanistic insights into endothelial dysfunction in PCOS and suggest potential implications for increased cardiovascular risk in affected individuals.

## Introduction

Polycystic ovary syndrome (PCOS) is one of the most common endocrine disorders, affecting 5–10% of reproductive-aged women (1). The diagnosis of PCOS requires the presence of at least two of the following characteristic features: clinical and/or biochemical hyperandrogenism (HA), chronic anovulation (AO), and polycystic ovarian morphology (PCOM) on ultrasonography, according to the Rotterdam criteria (1). Several studies have revealed evidence of endothelial cell (EC) dysfunction among women with PCOS and have attempted to provide further linkage between PCOS and long-term cardiovascular consequences. Significantly elevated circulatory markers of oxidative stress, endothelial damage, and inflammation, including malondialdehyde, N^G^, N^G^-dimethyl-l-arginine, high-sensitivity C-reactive protein, and platelet/endothelial cell adhesion molecule-1 (PECAM1) have been noted in women with PCOS (2, 3). Impaired vascular structure and endothelial function manifested by attenuated flow-mediated dilation and increased arterial intima-media thickness were also noted in lean (3, 4) and obese PCOS women (5).

Although accumulating evidence shows that patients with PCOS present with signs of EC dysfunction, the underlying pathophysiology remains unclear. HA is one of the most common features of PCOS and is closely related to its development and progression (6). Numerous clinical studies have revealed that HA is an independent risk factor for metabolic complications in women with PCOS, including obesity, insulin resistance (IR), type II diabetes mellitus (T2DM), hypertension (HTN), and atherosclerosis (7–10). In a case-control study performed by Usselman et al. (11), endothelin-1-induced nitric oxide (NO) production and microvascular dilatation were attenuated among lean, insulin-sensitive, hyperandrogenic PCOS women, suggesting that independent of obesity and IR, androgen itself contributes to EC dysfunction in PCOS. While most clinical observations and experimental studies have suggested detrimental cardiovascular effects of androgen excess in PCOS, the cardiovascular effects of androgen are complicated and vary in physiological and pathological conditions (12). Endogenous androgens promote angiogenesis and ischemia-induced vascular repair in a sex-dependent manner through androgen receptor–mediated activation of VEGF and PI3K–Akt signaling, conferring vascular regenerative advantages in males (13). However, the precise molecular mechanisms by which this complex androgen signaling mediates vascular biology in female and the EC dysfunction in PCOS remain largely unknown. In women with PCOS, there is evidence of enhanced 5α-reductase activity in peripheral tissues, which facilitates the local conversion of testosterone into dihydrotestosterone (DHT), the more potent androgenic metabolite (14). Elevated DHT levels have been clinically linked to more severe metabolic disturbances and increased cardiovascular risk factors in this population. To further elucidate the direct impact of potent androgens on the vasculature, DHT was employed as the primary androgenic stimulus in our model. DHT is a potent, non-aromatizable androgen with a significantly higher affinity for the androgen receptor (AR) compared to testosterone (15). By using DHT, we can specifically isolate AR-mediated signaling pathways and exclude the confounding effects of estrogen receptor activation that might occur via the aromatization of testosterone into estradiol.

To address this significant knowledge gap, we leveraged induced pluripotent stem cell (iPSC)-derived ECs. The use of iPSC-derived ECs provides a renewable source of ECs that overcomes the major limitations of traditional primary EC models, such as short lifespan and donor variability. Crucially, to ensure our model captures the intrinsic pathophysiology of the disease, we specifically established iPSCs from patients with phenotype A PCOS (fulfilling all three items of the Rotterdam criteria) (16, 17). By focusing on this “full-blown” phenotype, which carries the highest risk of endothelial dysfunction and metabolic aberrations, we aimed to minimize phenotypic heterogeneity and maximize the resolution of disease-specific molecular defects. Using this robust patient-specific platform, we sought to provide mechanistic clarity on androgen-mediated endothelial changes in PCOS. Many vascular diseases, such as pulmonary arterial disease, HTN, T2DM, cardiomyopathies, and calcified aortic valvular disease, have been studied using patient-specific iPSC-derived ECs as effective platforms (18–20). The differentiation protocol used was a monolayer culture with timed additions of molecules and growth factors, modified from Harding’s publication (21). Single-cell RNA sequencing (scRNA-seq) was used to characterize heterogeneous populations of patient-specific iPSC-derived ECs, and to verify the potential mechanisms of EC dysfunction in PCOS. The modulating roles of androgens on the proliferation and function of ECs in PCOS were further elucidated in the present study. We hypothesized that androgens, such as dihydrotestosterone (DHT), exacerbate vascular dysfunction in PCOS by dysregulating key cellular processes, including the cell cycle, vascular endothelial growth factor (VEGF) signaling, and angiogenesis, in iPSC-derived ECs.

## Materials and methods

### Derivation of PCOS and control iPSCs

Six human iPSCs were used in the present study, including three phenotype A PCOS iPSCs (P1, P5, and P7) and three control iPSCs (N1, N7, and N8). The establishment of iPSC was approved by the Research Ethics Committee of the National Taiwan University Hospital (200907056R), and all participants provided informed consent before enrollment. The patients were diagnosed with PCOS using the Rotterdam criteria with fulfillment of all three items: (1) amenorrhea or oligomenorrhea (fewer than eight spontaneous menstrual cycles per year for at least three years before enrollment); (2) biochemical HA (serum total testosterone level ≥0.8 ng/mL), and/or clinical HA, including hirsutism and alopecia; and (3) PCOM on ultrasonography (1). The demographic and major clinical data of the three PCOS patients are shown in **Supplementary Table 1**. The BMI and age of the three control subjects are also shown in **Supplementary Table 1**. All three PCOS patients were hyperandrogenic and obese (BMI>30). Two iPSC lines (P1 and N1) were reprogrammed from human skin fibroblasts by our group (22). The control N7 iPSC line (BCRC number: SC81036), which was purchased from the Bioresource Collection and Research Center (BCRC, Taiwan), was reprogrammed from human peripheral blood mononuclear cells (PBMCs). The N8, P5, and P7 iPSC lines were reprogrammed from PBMCs using the CytoTune TM-iPS 2.0 Sendai reprogramming kit, according to the manufacturer’s instructions (Thermo Fisher Scientific, Waltham, MA, USA; catalog number A16517). On day 21 post-transduction, embryonic stem cell (ESC)-like cell colonies were picked into murine embryonic fibroblast (MEF) feeders (Thermo Fisher Scientific, Waltham, MA, USA; catalog number A34180). The culture protocols for iPSCs were modified as previously described (23). Thus, iPSCs were continuously maintained on MEF feeders in serum-free medium (ReproCELL ES cell medium, Kanagawa, Japan; catalog number RCHEMD001), supplemented with 10 ng/mL basic fibroblast growth factor (bFGF) (R&D systems, Minneapolis, MN, USA; catalog number 233-FB-025/CF). The medium was changed every day. The cells were split weekly using 30-gauge insulin needles (Terumo Syringe, Tokyo, Japan; catalog number NN3025R) as previously described.

### Differentiation of patient-specific iPSCs towards ECs

Endothelial cellular differentiation protocols were modified from a previous study (21). Before differentiation, iPSCs were manually split into small clumps and plated onto human embryonic stem cells (hESC)-qualified Matrigel (Corning Inc., Corning, NY, USA; catalog number 354277)-coated 6-well plate in MEF-conditioned medium with an additional 10 ng/mL bFGF. On day 0, the cells were treated with 6 μM CHIR99021 (Tocris Bioscience, Avonmouth, UK; catalog number 4423) in StemDiff APEL medium (STEMCELL Technologies, Vancouver, BC, Canada; catalog number 05210). After three days, 25 ng/mL bone morphogenetic protein 4 (BMP4) (R&D Systems, Minneapolis, MN, USA; catalog number 314-BP), 10 ng/mL bFGF, and 50 ng/mL VEGF (R&D Systems, Minneapolis, MN, USA; catalog number 293-VE) were added to StemDiff APEL medium for two days. Next, the cells were dissociated with Accutase (Innovative Life Technologies, San Diego, CA, USA; catalog number 00-4555-56) and seeded onto 1% gelatin (Sigma-Aldrich, St. Louis, MO, USA; catalog number G1393)-coated cell culture dishes in EC growth medium MV2 (ECGM-MV2) (PromoCell, Heidelberg, Germany; catalog number C-22022) supplemented with 50 ng/mL VEGF. The ECGM-MV2 was changed every two days for EC generation. The iPSC-derived ECs were split every 4-6 days for passage. iPSC-derived ECs used in this experiment were at passage 2-5. In some experiments, iPSC-derived ECs were incubated with different concentrations (1, 10 or 100 nM) of DHT (Sigma-Aldrich, St. Louis, MO, USA; catalog number D-073-1ML) after overnight starvation in 1% fetal bovine serum (FBS) (Thermo Fisher Scientific, Waltham, MA, USA; catalog number A5670701) medium for the indicated time. The concentrations of DHT used in the functional assays (1, 10, and 100 nM) were selected to encompass the full spectrum of biological and pathological androgenic environments. Specifically, 1 nM DHT represents the upper limit of the physiological range in healthy females, while 10 nM mimics the physiological levels found in biological males (24, 25). The highest concentration, 100 nM, was utilized to stimulate the local hyperandrogenic microenvironment characteristic of PCOS tissues, such as the endometrium (26). Previous investigations using human GC-like cells (KGN) demonstrated that treatment with 100 nM DHT effectively mimics the deleterious impact of severe hyperandrogenism (27). Given the constraints of short-term *in vitro* stimulation (24 hr), this higher concentration is necessary to model the cumulative pathological impact of chronic androgen exposure on endothelial function, consistent with previously established models (28).

### RNA extraction, cDNA preparation, and quantitative PCR

Total RNA was harvested from iPSC-derived ECs, including two control (N1 and N7) and two PCOS (P1 and P5) lines for assessing intrinsic gene expression. For DHT treatment studies, three control (N1, N7, and N8) and three PCOS (P1, P5, and P7) iPSC-derived ECs were treated with DHT at concentrations of 0, 1, 10, and 100 nM for 24 hr pior to RNA isolation. 4x10^5/^well cells were seeded in 1% gelatin-coated 6-well plates and grown to 80-90% confluence prior to RNA extraction. Experiments were performed in three independent biological replicates. Extraction was performed using TRIzol reagent (Invitrogen, Waltham, MA, USA; catalog number 15596018), following the manufacturer’s instructions. Reverse transcription was performed using a Maxima First Strand cDNA Synthesis Kit (Thermo Scientific, Waltham, MA, USA; catalog number K1641). Thus, 1-10 μg of total RNA was treated with 1 μL of 10× DNase buffer and 1 μL of amplification grade DNase I in a 20 μL reaction. The reaction was terminated by adding 1 μL of DNase stop buffer (Promega, Madison, WI, USA; catalog number M6106). Reverse transcription was performed at 25 °C for 10 min and at 50 °C for an hour, and the reaction was terminated by incubation at 85 °C for 5 min. Samples of cDNA were then aliquoted and stored at -20 °C. Quantitative PCR (qPCR) reactions were performed with the Fast SYBR Green Master Mix (Thermo Scientific, Waltham, MA, USA; catalog number 4385612) on the Applied Biosystems StepOne Plus system (Applied Biosystems, Carlsbad, CA, USA). One microliter of diluted cDNA and 100 μM of selected primers were used at a total volume of 2 μL in a 20 μL qPCR reaction. The qPCR procedure was initiated for 2 min at 95 °C, followed by 40 cycles of 15 s at 95 °C, 30 s at 55 °C, and 30 s at 72 °C. The expression levels of the target genes were calibrated using eukaryotic translation elongation factor 1 alpha (eEF1α1) and the data were analyzed using the ABI 7500 Fast Real-Time PCR system. The sequences of qPCR probes are listed in **Supplementary Table 2**. Quantification of all samples using the software was calculated from the cycle threshold (CT), and relative fold changes were calculated using the 2^- ΔΔCT^ method.

### Flow cytometry analysis and Immunofluorescence

iPSC-derived ECs from two control (N1 and N7) and two PCOS (P1 and P5) lines were seeded onto 1% gelatin-coated 6 cm dishes at a density 1X10^6^ cells per dish. Upon reaching 80-90% confluency, the cells were dissociated using Accutase to prepare single-cell suspensions. The cells were fixed with 4% paraformaldehyde (Thermo Scientific, Waltham, MA, USA; catalog number J61899.AK) and blocked with 5% bovine serum albumin (BSA) (Sigma-Aldrich, St. Louis, MO, USA; catalog number A9418). General endothelial surface markers such as kinase insert domain receptor (KDR)/VEGF receptor 2 (VEGFR2), CD31/PECAM1, or CD144/vascular endothelial cadherin (VE-Cadherin) were used to determine iPSC-derived ECs (29). CD31^+^CD144^+^ double positive cells differentiated from iPSCs were characterized as endothelial lineage-committed mature ECs. For flow cytometry, PE-conjugated mouse anti-human CD31 (BD Biosciences, Milpitas, CA, USA; catalog number 560983) and FITC-conjugated mouse anti-human CD144 (BD Biosciences, Milpitas, CA, USA; catalog number 560874) antibodies were used at a 1:10 dilution. Data were analyzed by a two-step gating procedure using CellQuest software (BD Biosciences, Milpitas, CA, USA). First, forward-scatter versus side-scatter gates were set to exclude dead cells. Second, iPSC-derived ECs were identified by the CD31-PE and CD144-FITC combination. For each sample, a minimum of 10,000 total cellular events were acquired on the flow cytometer (BD FACSCalibur). For immunofluorescence (IF) analysis, cells from two control (N1 and N7) and two PCOS (P1 and P5) iPSC-derived ECs were seeded at a density of 2.5 x 10^5^ cells per well onto 1% gelatin-coated 4-well glass chamber slides (Millicell EZ Slide; Merk KGaA, Darmstadt, Germany; catalog number PEZGS0416). Cells were fixed with 4% paraformaldehyde for 15 minutes, followed by permeabilization with 0.3% Triton X-100 (Thermo Scientific, Waltham, MA, USA; catalog number A16046.AE) for 10 minutes at room temperature. The von Willebrand Factor (vWF) (Abcam, Cambridge, MA, UK; catalog number ab6994) was used at a 1:50 dilution. The secondary antibody, Alexa Fluor 594 donkey anti-sheep (Abcam, Cambridge, MA, UK; catalog number ab150180), was used at a 1:500 dilution. Hoechst 33342 (R&D Systems, Minneapolis, MN, USA; catalog number 5117) was used for nuclear staining (blue). Experiments were performed in three independent biological replicates. Images were captured at 200x magnification and were examined by using an inverted fluorescence microscope (ECLIPSE TE2000-U, Nikon, Tokyo, Japan). Fluorescence intensity was analyzed using Fiji for the Mac OS X version of ImageJ.

### Dil-Ac-LDL uptake assay

Cells from two control (N1 and N7) and two PCOS (P1 and P5) iPSC-derived ECs were seeded at a density of 2.5 x 10^5^ cells per well onto 1% gelatin-coated 4-well glass chamber slides. After 1% FBS medium starvation overnight, the cells were incubated with 10 μg/ml Alexa Fluor 594-conjugated acetylated low-density lipoprotein (Invitrogen, Waltham, MA, USA; catalog number L35353) at 37 °C for 4 hr. Hoechst 33342 was used for nuclear staining (blue). The cells were washed with PBS three times to remove the excess stain. Experiments were performed in three independent biological replicates. Images were captured at 400x magnification and were examined by using an inverted fluorescence microscope (ECLIPSE TE2000-U). Fluorescence intensity was analyzed using Fiji for the Mac OS X version of ImageJ.

### Western blot analysis

1-1.5 x 10^7^ cells from the two control (N1 and N7) and two PCOS (P1 and P5) iPSC-derived ECs were lysed in RIPA lysis buffer (Millipore, Billerica, MA, USA; catalog number 20-188). The buffer was supplemented with Halt Protease & Phosphatase inhibitor cocktail (Thermo Scientific, Waltham, MA, USA; catalog number 788443) to prevent protein degradation. Western blot analysis was performed as previously described (23). Protein concentration was determined by the Pierce BCA Protein Assay Kit (Thermo Scientific, Waltham, MA, USA; catalog number A65453). Samples with 30 μg of total proteins were electrophoretically separated on a 12% SDS-polyacrylamide gel and transferred onto PVDF membranes (Millipore, Billerica, MA, USA; catalog number IPVH09120). All primary antibody incubations were performed at 4 °C overnight at the following dilutions: rabbit anti-Cyclin D1 (Abcam, Cambridge, MA, USA; catalog number ab16663), 1:1000; rabbit anti-Cyclin E1 (Abcam, Cambridge, MA, USA; catalog number ab33911), 1: 200; mouse anti-p21 (Invitrogen, Waltham, MA, USA; catalog number MA5-14949), 1:500; rabbit anti-p53 (Abcam, Cambridge, MA, USA; catalog number ab131442), 1:1000; rabbit anti-actin (Abcam, Cambridge, MA, USA; catalog number ab8227), 1:500. After the secondary antibodies reaction, the signal was detected using ECL (Pierce Western Blot Signal Enhancer) (Thermo Scientific, Waltham, MA, USA; catalog number 21050). Chemiluminescent signals were captured using a MultiGel-21 imaging system (Topbio, New Taipei City, Taiwan). The signals of the bands on blots were measured with Fiji for the Mac OS X version of ImageJ. Relative protein expression levels were determined by the density ratio of the target protein versus housekeeping protein and normalized to control. Experiments were performed in three independent biological replicates.

### Tube formation assay

Three control (N1, N7, and N8) and three PCOS (P1, P5, and P7) iPSC-derived ECs pre-treated DHT (1, 10 or 100 nM) overnight and then seeded into growth factor-reduced Matrigel-coated 96-well plates (Corning Inc., Corning, NY, USA; catalog number 354230) at density of 1.5 x 10^4^ cells per well. Cells were cultured in ECGM-MV2 under continuous treatment of DHT exposure. After 6 hr, tube formation was captured at 40x magnification using an inverted microscope (ECLIPSE TE2000-U). Experiments were performed in three independent biological replicates. Total tubal length was calculated with the Angiogenesis Analyzer using Fiji plug-in for the Mac OS X version of ImageJ.

### Wound healing assay

Three control (N1, N7, and N8) and three PCOS (P1, P5, and P7) iPSC-derived ECs were seeded onto 1% gelatin-coated 6-well plates at a density of 1 x 10^6^ cells per well in ECGM-MV2 to reach 80-90% confluency. Three vertical and three horizontal scratches were created in each well using a 200 μl pipette tip, followed by two wash with PBS to remove cellular debris. The cells were then cultured in ECGM-MV2 supplemented with 1% FBS and treated with varying concentrations of DHT (1, 10, or 100 nM) for 24 hr. Experiments were performed in three independent biological replicates. Images of the scratched areas were captured at 0 and 24 hr post-scratching at 40x magnification using an inverted microscope (ECLIPSE TE2000-U). The wound closure area was quantified using Fiji for the Mac OS X version of ImageJ.

### Cell proliferation assay

Three control (N1, N7, and N8) and three PCOS (P1, P5, and P7) iPSC-derived ECs were seeded onto 1% gelatin-coated 96-well plates at a density of 1.5 x 10^4^ cells per well. After overnight incubation, the cells were treated with DHT at various concentrations (0, 1, 10, or 100 nM) in ECGM-MV2 supplemented with 1% FBS for 24 hr. Viable cells were quantified by trypan blue exclusion and counted on a Luna automated cell counter (Biocat GmbH, Heidelberg, Germany). Cell proliferation assay was performed with the EZ-5-bromo-2’-deoxyuridine (BrdU) Kit (TonboTM A Cytek Brand, San Diego, CA, USA; catalog number TNB-6600-KIT) following the manufacturer’s instructions. Briefly, three control and three PCOS iPSC-derived ECs were seeded onto 1% gelatin-coated 6 cm dishes at a density of 1.5 x 10^6^ cells per dish. After overnight incubation, the medium was replaced with ECGM-MV2 supplemented with 1% FBS containing different concentrations of DHT (0, 1, 10, or 100 nM) for 24 hr. During finial stages of treatment, BrdU was added to the culture medium. EZ-BrdU-labeled iPSC-derived ECs were fixed with 70% ethanol (Sigma-Aldrich, St. Louis, MO, USA; catalog number 108543) overnight in -20 °C. Fixed cells were washed in wash buffer (1% BSA, 0.25% Triton-X and 1x PBS). Cells were resuspended with DNA denature buffer (3N HCl, 0.5% Tween-X and 1x PBS) and incubated for 30 minutes at room temperature. Denatured cells were then neutralized with neutralization buffer (0.1M sodium borate). Cells were incubated with BrdU antibody solution for 1 hr at room temperature in the dark. Propidium Iodide (PI)/RNase A solution was added to cells for DNA counter staining and incubated for 30 minutes at room temperature in the dark and analyzed the cells using a FACS machine directly. For each experimental condition, a minimum of 10,000 total cellular events were acquired on the flow cytometer. Single-cell events were distinguished and gated based on the linear relationship between PI staining of FL2-A vs. FL2-H parameters. Events deviating from the diagonal line, representing cell aggregates, were strictly excluded from the final analysis to ensure accurate DNA content measurements. The cell cycle phases were defined on the bivariate plot of BrdU incorporation (FL1-H, log scale) versus total DNA content (FL2-A, linear scale). Cells with diploid DNA content (FL2-A peak) and minimal or no BrdU incorporation (FL1-H negative) were identified as G1 phase. Cells with DNA content between the G1 and G2/M peaks and demonstrating significant BrdU incorporation (FL1-H positive) were identified as S phase. Cells with tetraploid DNA content and BrdU incorporation generally lower than S phase were identified as G2/M phase. Experiments were performed in three independent biological replicates. Data represent the percentage of cells in each cell cycle phase.

### Droplet-based single-cell RNA-sequencing

For the 10x Genomics scRNA-seq assay, two control (N1 and N7) and two PCOS (P1 and P5) iPSC-derived ECs were cultured in 1% gelatin-coated 10 cm dishes. Upon reaching 80-90% confluency, the cells were dissociated using Accutase to prepare single-cell suspensions. For each line, the total cell number was confirmed to exceed 1 x 10^7^ with viability of > 90%. Dead cells were removed using the MACS Dead Cell Removal kit (Miltenyi Biotec, Bergisch Gladbach, Germany; catalog number 130-090-101), following the manufacturer’s instructions. Live cells were resuspended in PBS, containing 0.04% BSA and adjusted to 1600-1900 cells/μL. Approximately 10,000 cells from each sample were loaded as gel beads in emulsion (GEMs, 10x Genomics, Pleasanton, CA, USA) into one channel of Single Cell 3’ Chips v3 (10x Genomics, Pleasanton, CA, USA) using a 10x Chromium controller (10x Genomics, Pleasanton, CA, USA). Single cells were encapsulated in gel beads that contained (1) sequencing adapters and primers; (2) a 14-base pair barcode drawn from approximately 750,000 designed sequences for indexing GEM; (3) a 10-base pair randomer to index molecules (unique molecular identifier, UMI); and (4) an anchored 30-base pair oligo-dT to prime polyadenylated RNA transcripts. The cDNA was synthesized and amplified in each gel bead using a Bio-Rad C1000 Touch™ Thermal Cycler, and then cleaned up by post GEMs-RT following the manufacturer’s protocol. cDNA was quantified using the Qubit™ dsDNA HS Assay Kit (Invitrogen, Waltham, MA, USA; catalog number Q32851). Illumina sequencing libraries were constructed using the Agilent Bioanalyzer High-Sensitivity DNA kit (Agilent Technologies, Taipei, Taiwan; catalog number 5067-4626). Transcriptome libraries were sequenced using the Illumina NovaSeq 6000 S1 Sequencing System with 150-base paired-end reads.

### ScRNA-seq data processing and analysis

CellRanger single-cell software suite (v3.1.0) was used to align reads to the human reference genome GRCh38-3.0.0 and to extract the cell and unique molecular identifier (UMI) barcodes. Scater (v1.14.6) was used to identify outliers based on library size, number of expressed genes, and mitochondrial proportion. If cells had a value greater or lower than three times the median absolute deviation (MAD) for each metric, they were removed. The Scrublet was used for cell doublet detection, and the cells predicted as doublets were removed.

### Statistical analysis

Statistical analyses were conducted using GraphPad Prism 10 (GraphPad Software, San Diego, CA, USA). Data are expressed as mean ± standard deviation (SD). For comparisons between two independent groups, such as the basal protein expression levels in control versus PCOS groups analyzed by Western blot, an unpaired t-test was utilized. To evaluate differences across multiple groups or variables, one-way or two-way analysis of variance (ANOVA) was performed as appropriate. Specifically, one-way ANOVA followed by Tukey’s *post-hoc* test was used to compare gene expression levels among the four iPSC-derived ECs, while a two-way ANOVA with Tukey’s multiple comparisons test was employed to analyze the distribution of cell cycle phases across these lines. Furthermore, the dose-dependent effects of DHT treatment were analyzed to assess its impact on cellular proliferation and mRNA expression. For the analysis of proliferation rates and mRNA levels within the control or PCOS groups, a one-way ANOVA followed by Dunnett’s *post-hoc* test was applied to compare each DHT concentration specifically against the 0 nM control. Similarly, a two-way ANOVA followed by Dunnett’s test was used to evaluate the impact of various DHT concentrations on the cell cycle phases of each individual cell line, with comparisons made solely against the untreated control (0 nM) for each respective phase. In all analyses, a p-value < 0.05 was considered to indicate statistical significance.

In scRNA-seq data analysis, Seurat (4.1.1) was used to normalize the raw count data, identify highly variable genes, scale expression values, integrate samples, regress out the effect of the cell cycle, decrease data dimensions, and identify cell clusters and differentially expressed genes (30). Highly variable genes (HVGs) were identified using the FindVariableFeatures function in Seurat with variance-stabilizing transformation (vst) method. Cell cycle phase scores were calculated using the CellCycleScoring function implemented in Seurat, based on the canonical S phase and G2/M phase gene sets provided by Seurat. Cell cycle scores were subsequently regressed out during data scaling to minimize cell cycle–associated transcriptional variation in downstream analyses. Principal component analysis (PCA) was performed based on the 2000 HVGs, and the first 20 principal components (PCs), determined by inspection of the elbow plot and the proportion of variance explained, were used for t-distributed stochastic neighbor embedding (t-SNE) dimensionality reduction and graph-based clustering. A k-nearest neighbor (kNN) graph was constructed in the space of the first 20 PCs using default parameters implemented in Seurat, and was subsequently converted into a shared nearest neighbor (SNN) graph for downstream clustering. Clusters were identified by applying the Louvain algorithm to the SNN graph, with a resolution parameter of 0.5. Differential expression genes between cell clusters, as well as between PCOS and control samples within the same cell cluster, were identified using the FindAllMarkers functions in Seurat with MAST method. Genes with statistically significant differential expression (FDR < 0.05) were used for downstream functional enrichment analyses. Overrepresentation analysis was performed using the enricher function implemented in clusterProfiler on the gene sets from MSigDB (v7.4).

## Results

### Differentiation of endothelial cells from PCOS and control iPSCs

The ECs were successfully differentiated from iPSC lines from three control participants and three patients with PCOS. After passage, both control and PCOS iPSC lines generated CD31^+^CD144^+^ double-positive ECs with high differentiation efficiency (up to 90% in each population) (**Supplementary Figure 1A**). They also expressed mature EC marker vWF (**Supplementary Figure 1B**) and demonstrated comparable abilities to uptake LDL and form tubes on Matrigel (**Supplementary Figures 1C, D**). These data indicate that ECs were successfully differentiated without sorting, and there was no significant difference in the differentiation efficiency between the PCOS and control groups.

### Characterization of PCOS and control iPSC-derived ECs by single-Cell RNA sequencing

**Figure 1:**
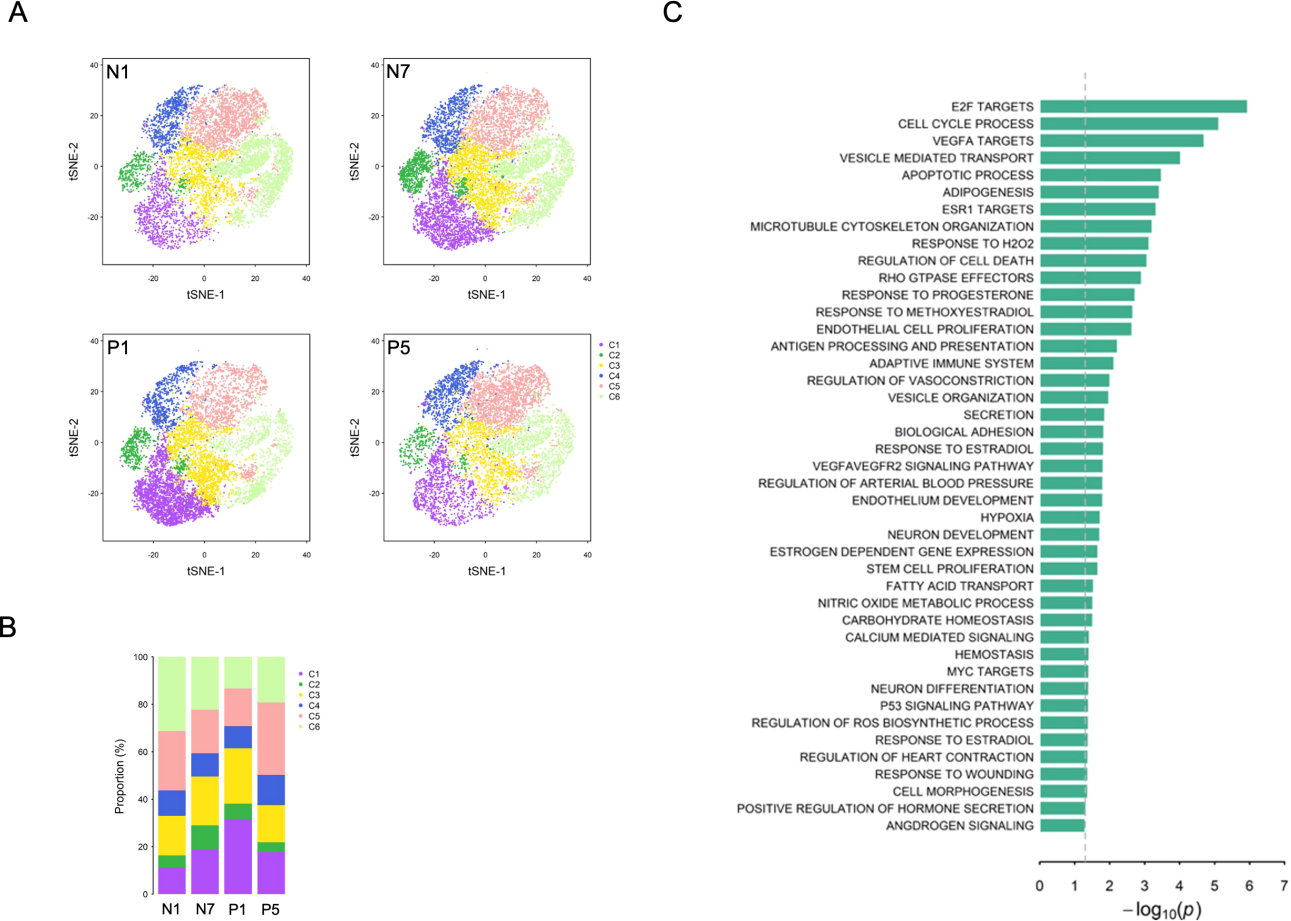
Single cell RNA sequencing analysis of induced pluripotent stem cell (iPSC)-derived endothelial cells (ECs) from control and PCOS samples. **(A)** t-distributed stochastic neighbor embedding (t-SNE) visualization of iPSC-derived ECs from two control (N1 and N7) and two PCOS (P1 and P5) samples. Six distinct clusters (C1-C6) were identified based on transcriptomic similarity independent of sample origin. **(B)** Bar plot showing the proportion of cells assigned to each of the six clusters in each sample. **(C)** Significantly enriched pathways identified based on differentially expressed genes between PCOS and control samples across the six cell clusters. Bar length represents the enrichment significance expressed as -log10 (p-value). The differentially expressed genes were showed in **Supplementary Figure 3**.

To investigate the pathogenesis of EC dysfunction in PCOS women, scRNA-seq was performed on iPSC-derived ECs from two PCOS and two control subjects using the 10X Genomics Chromium platform. On average, 11,937 (range 9,713-13,440) cells were profiled per sample (**Supplementary Table 3**). After normalization and integration, cells were visualized using t-SNE, and six distinct cell clusters (C1-C6) were identified based on transcriptomic similarity (**Figure 1A**). Canonical EC marker genes, including PECAM1, Fms-related receptor tyrosine kinase 1 (FLT1), cadherin-5 (CDH5), endothelial cell surface expressed chemotaxis and apoptosis regulator (ECSCR), CD34, ETS transcription factor (ERG), and vWF, were expressed in most iPSC-derived ECs (**Supplementary Figure 2A**). To assess the purity of our iPSC-derived EC population, we examined the expression of genes representing major potential contaminating lineages. Markers of neural/ectodermal marker (NCAM1), hematopoietic/immune markers (CD79A, CD19, CD14, integrin subunit alpha M (ITGAM), and HLA-DR clusters), and fibroblastic markers [collagen type I alpha 1 chain (COL1A1) and collagen type I alpha 2 chain (COL1A2)] were either absent or detected in less than 2% of cells. To further exclude contamination by mural cells, we analyzed markers for pericyte (platelet-derived growth factor receptor beta (PDGFRB): 0.06%, chondroitin sulfate proteoglycan 4 (CSPG4): 0.03%) and smooth muscle cell (actin alpha 2 (ACTA2): 16.8%, myosin heavy chain 11 (MYH11): 0.46%, desmin (DES): 0%). Despite the presence of ACTA2 in a subset of cells, the absence of mature contractility markers (MYH11 and DES) and pericyte-specific markers (PDGFRB and CSPG4) indicates a lack of mature mural cell contamination. These results collectively demonstrate high endothelial commitment in the cells generated in this study (**Supplementary Figure 2B**). Lineage analysis further revealed that majority of iPSC-derived ECs exhibited an arterial identity, characterized by expression of neuropilin 1 (NRP1), ephrin B2 (EFNB2), and notch receptor 1 (NOTCH1). In contrast, markers of venous EC [EPH receptor B4 (EPHB4), nuclear receptor subfamily 2 group F member 2 (NR2F2), and notch receptor 4 (NOTCH4)] and lymphatic EC [prospero homeobox 1 (PROX1) and podoplanin (PDPN)] were minimally expressed or undetectable (**Supplementary Figure 2B**).

**Figure 2:**
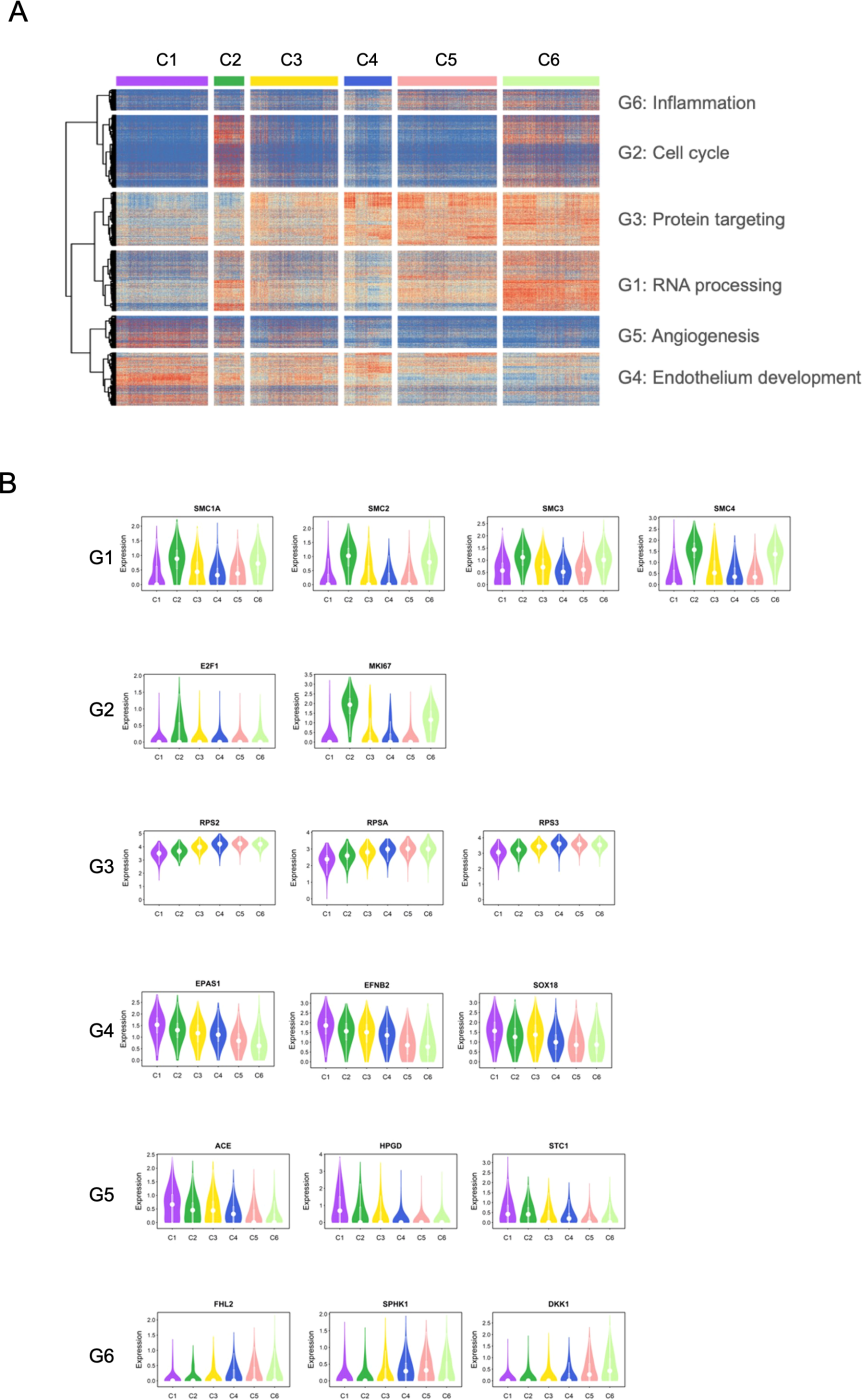
Functional annotation and characterization of cell clusters. **(A)** Heatmap analysis of differentially expressed genes (DEGs) between cell clusters. DEGs were categorized into six functional groups (G1-G6) based on hierarchical clustering. G2 (cell cycle) and G1 (RNA processing) genes are predominantly expressed in C2 and C6, identifying these clusters as the proliferative hubs of the population. Other groups represent functional states such as protein targeting (G3), endothelium development (G4), angiogenesis (G5), and inflammation (G6). **(B)** Representative DEGs across clusters. Violin plots show the expression levels of key markers for each functional group. In G1, structural maintenance of chromosomes protein (SMC) family genes (SMC1A, SMC2, SMC3, and SMC4 genes) are related to RNA processing; In G2, E2F transcription factor 1 (E2F1) and (marker of proliferation Ki-67) MKI67 genes are related to cell cycle; in G3, ribosomal protein S2 (RPS2), ribosomal protein SA (RPSA), and ribosomal protein S3 (RPS3) genes are related to protein targeting; in G4, endothelial PAS domain-containing protein 1 (EPAS1), ephrin B2 (EFNB2), and SRY-Box transcription factor 18 (SOX18) genes are related to endothelium development; In G5, angiotensin-converting enzyme (ACE), 15-hydorxyprostaglandin dehydrogenase (HPGD), and stanniocalcin 1 (STC1) genes are related to angiogenesis; and in G6, four and a half LIM domains 2 (FHL2), sphingosine kinase 1 (SPHK1), and dickkopf WNT signaling pathway inhibitor 1 (DKK1) genes are related to inflammation. G1 and G2 (proliferation): SMA1A-4, E2F1, and MKI67 are highly enriched in C2 (cycling ECs) and C6 (active synthetic ECs). G4 and G5 (maturation and angiogenesis): EPAS1, EFNB2, and SOX18 are markers for C1 (maturing ECs) and C4 (angiogenic ECs). G6 (Signaling): FHL2 and SPHK1 characterize C5 (signaling/inflammatory ECs). These functional annotations reveal that the heterogeneity among clusters reflects dynamic functional states rather than distinct anatomical EC subtypes.

To characterize functional difference between EC clusters identified by scRNA-seq, we analyzed differentially expressed genes (DEGs) through pairwise comparison between cell clusters. As shown in **Figure 2A**, hierarchical clustering of DEGs did not reveal sharply segregated gene expression patterns across the six clusters. Instead, gene expression profiles exhibited substantial overlap among clusters, suggests that the identified clusters likely represent closely related and transitional endothelial states rather than discrete cell types. Markers of canonical EC and lineage exhibit similar pattern across the six cell clusters (**Supplementary Figures 2C, D**).

Based on co-expression pattern, DEGs were grouped into six functional modules associated with RNA processing (G1), cell cycle (G2), protein targeting (G3), endothelium development (G4), angiogenesis (G5), and inflammation (G6), respectively (**Figure 2A**). These modules showed graded and overlapping expression patterns across clusters rather than binary, cluster-restricted activation. Consistent with the module-based clustering, clusters C2 and C6 showed relatively higher expression of genes belonging to the cell cycle- and RNA-processing-associated modules (G2 and G1) compared with other clusters (**Figure 2B**). In addition, C6 exhibited increased expression of representative genes from the protein targeting (G3) and inflammation (G6) modules, indicating coordinated activation of multiple transcriptional programs (**Figure 2B**). Clusters C1 showed higher expression of genes associated with endothelium development (G4) and angiogenesis-related processes (G5), with representative genes such as EFNB2, SOX18, and EPAS1 more prominently expressed in this cluster (**Figure 2B**). In contrast, clusters C3, C4, and C5 did not exhibit strong preferential enrichment for a single functional module. Instead, these clusters displayed mixed and intermediate expression patterns across multiple modules, consistent with overlapping transcriptional programs rather than dominance of a specific functional signature. Together, these results indicate that EC clusters are characterized by partially overlapping transcriptional programs represented by distinct co-expression modules, rather than by discrete, cluster-specific marker genes.

To compare the composition of cell cluster across sample, PCOS samples (P1 and P5) exhibit a reduced proportion of C2 (cycling state) and C6 (active synthetic/proliferative state) compared to control samples (N1 and N7) (**Figure 1B**). In addition, DEGs between PCOS and control samples were identified within each cell cluster (**Supplementary Figure 3**). Functional enrichment analysis revealed that DEGs were significantly enriched in cell-cycle- and proliferation-related pathways (**Figure 1C**). These results indicate that alterations in cell cycle–associated transcriptional programs are a prominent feature distinguishing ECs from PCOS and control subjects.

### Decreased cellular proliferation of iPSC-derived ECs in the PCOS group due to cell cycle arrest

**Figure 3:**
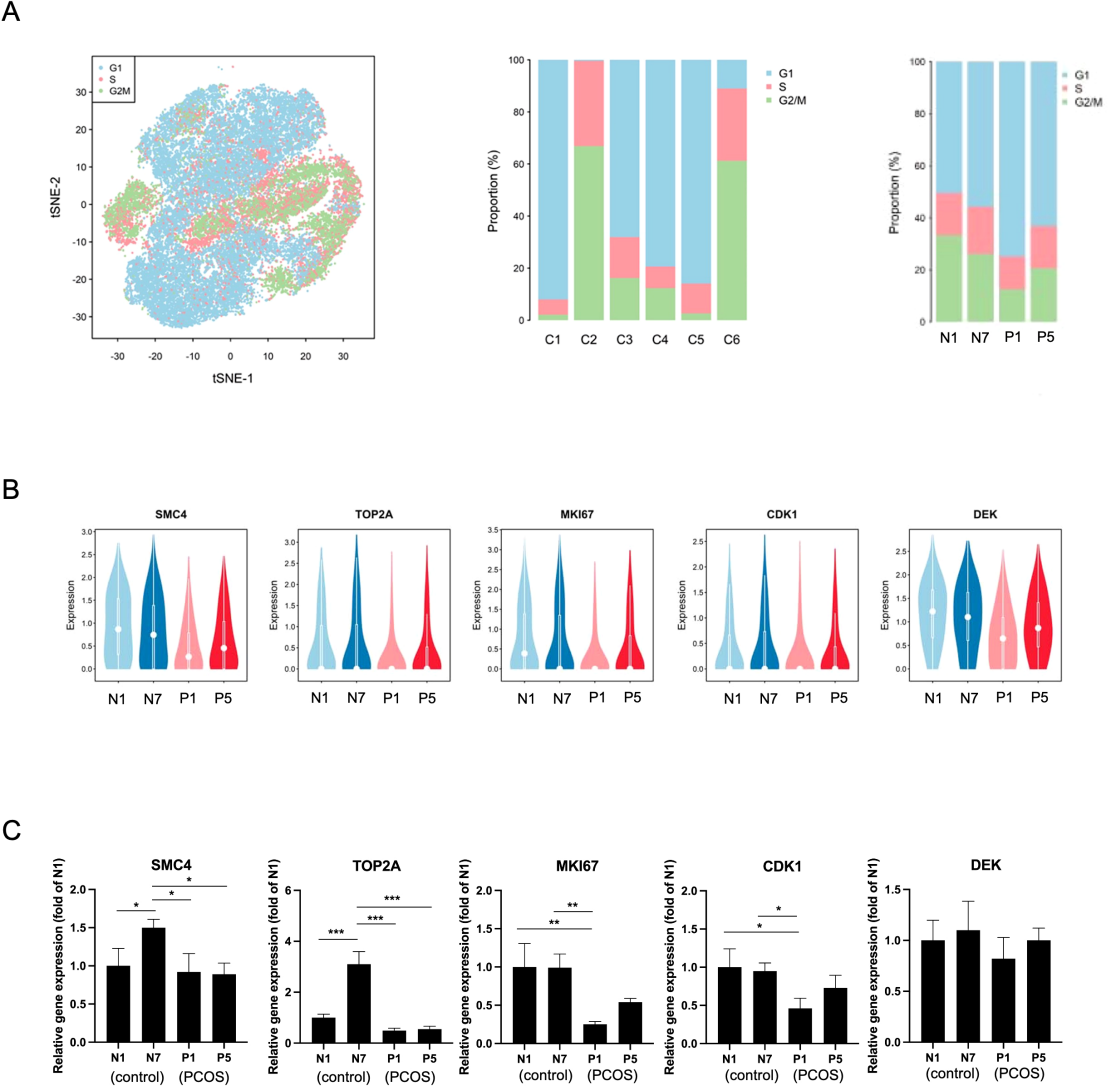
Impaired cell cycle progression and reduced expression of proliferative markers in PCOS iPSC-derived ECs. **(A)** Single-cell cell cycle phase distribution. (Left) t-SNE plot of all iPSC-derived ECs colored by cell cycle phase: G1 (blue), S (red), and G2/M (green). (Middle) Stacked bar plot showing the proportion of cell cycle phases within each cluster (C1-C6). C2 and C6 are identified as the primary proliferative clusters. (Right) A quantitative comparison of cell distribution is provided. An increased proportion of cells in the G1 phase, accompanied by a significant reduction in the S and G2/M populations, is observed in PCOS samples (P1 and P5). **(B)** Transcriptomic profiling of proliferative markers. The expression levels of key cell cycle genes, including SMC4, DNA topoisomerase II alpha (TOP2A), MKI67, cyclin-dependent kinase 1 (CDK1) and DEK proto-oncogene (DEK), are displayed via violin plots. A consistent downregulation of these genes is observed in the PCOS group, reflecting a systemic decrease in the molecular machinery required for cell cycle progression beyond the G1 phase. **(C)** Validation of gene expression. The scRNA-seq findings were further substantiated by Real-Time qPCR. Significant downregulations of SMC4, TOP2A, MKI67, CDK1 and DEK were confirmed in PCOS iPSC-derived ECs. While a lower mean expression of DEK is noted, no statistical significance was reached. Data are presented as relative fold change (mean ± SD); *P<0.05; **P<0.01; ***P<0.001. n=3 independent experiments.

Building on the observation that cell cycle-associated transcriptional programs distinguished endothelial cell (EC) clusters and differed between PCOS and control samples (**Figure 1**), we next examined cell cycle dynamics in iPSC-derived ECs in greater detail. Cell cycle phase assignment revealed differences in the distribution of G1, S, and G2/M phases across EC clusters and samples (**Figure 3A**). At the cluster level, clusters C2 and C6 contained a higher proportion of cells in S and G2/M phases compared with other clusters, whereas clusters C1, C3, C4, and C5 were predominantly composed of G1-phase cells. At the sample level, PCOS iPSC-derived ECs (P1 and P5) exhibited a reduced proportion of cells in S and G2/M phases compared with control samples (N1 and N7), indicating altered cell cycle phase distribution in PCOS ECs.

**Figure 4:**
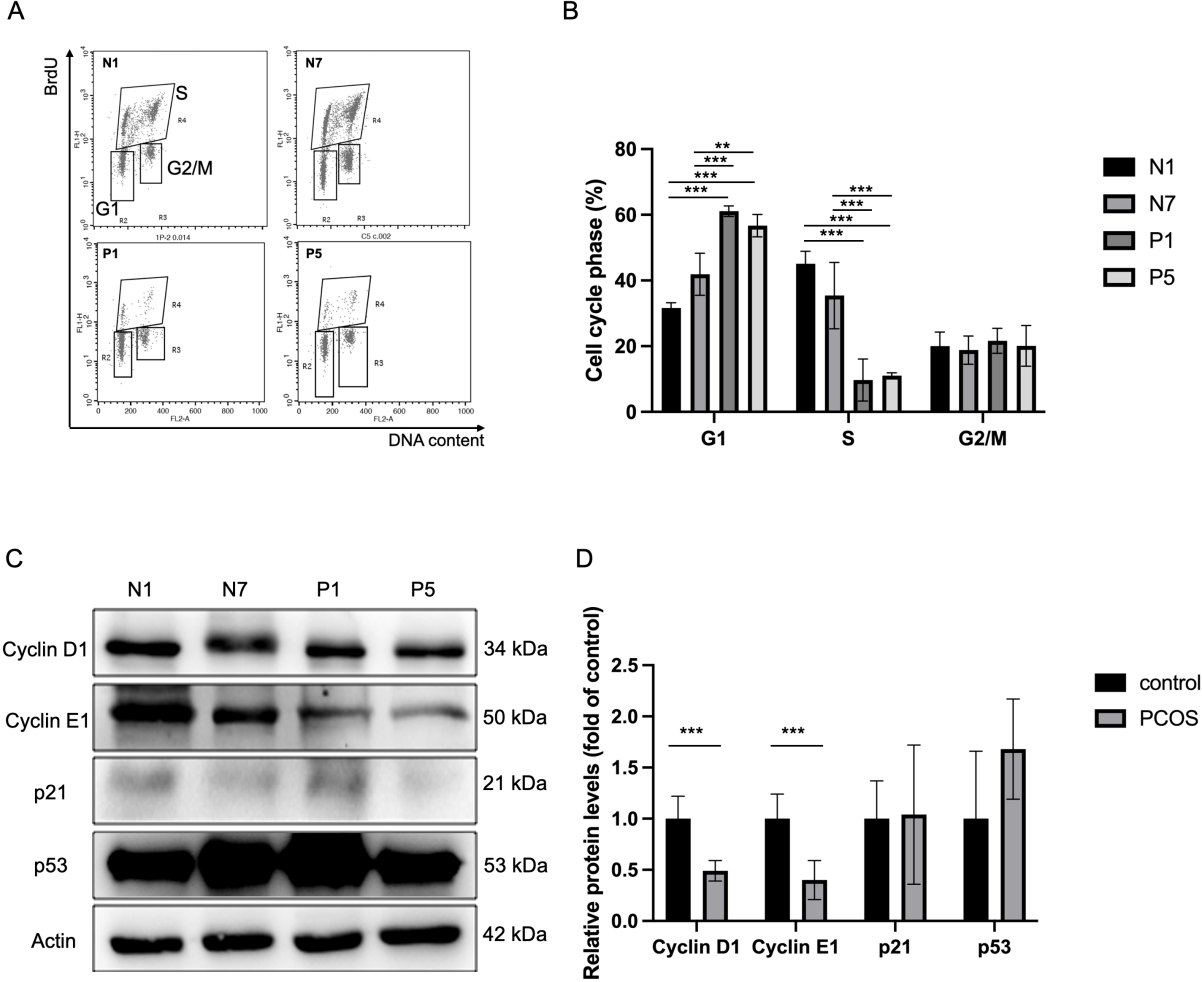
The Cell proliferation assay indicated fewer proliferating cells in PCOS iPSC-derived ECs than in control iPSC-derived ECs. Cell cycle distribution was analyzed with EZ-5-bromo-2’-deoxyuridine (BrdU) kit by flow cytometry **(A)**. **(B)** Quantification of iPSC-derived ECs in G1, S and G2/M phases by flow cytometry. There were significantly reduced S phase cells and accumulated G1 phase cells in PCOS iPSC-derived ECs compared with control iPSC-derived ECs. **(C, D)** The expression of cell cycle proteins was determined by Western blot. The expression of Cyclin D1 and Cyclin E1 are simultaneously lower in PCOS iPSC-derived ECs (P1+P5) compared to the control iPSC-derived ECs (N1+N7). There was no significant difference in the expression of other cell cycle regulators, p21 and p53, between the PCOS (P1+P5) and control (N1+N7) iPSC-derived ECs. Data are represented as mean ± SD (n=3 independent experiments). Grouped data, control (N1+N7) and PCOS (P1+P5), were used for comparison (n=6 independent experiments). *P<0.05; **P<0.01; ***P<0.001 indicate statistical significance.

Consistent with these observations, expression of canonical cell cycle-related genes was reduced in PCOS iPSC-derived ECs (**Figure 3B**). Key regulators of cell cycle progression and mitosis, including structural maintenance of chromosomes 4 (SMC4), DNA topoisomerase II alpha (TOP2A), marker of proliferation ki-67 (MKI67), cyclin-dependent kinase 1 (CDK1), and DEK proto-oncogene (DEK), showed lower expression levels in PCOS samples relative to controls. The expression of proliferation-related genes was confirmed by Real-Time RT-PCR (**Figure 3C**). SMC4, TOP2A, CDK1, and MKI67 showed significant higher expression in control iPSC-derived ECs than in PCOS iPSC-derived ECs. While DEK showed a lower mean expression in the PCOS group, this difference did not reach statistical significance (**Figure 3C**). Together, these results indicate that cell cycle–associated transcriptional activity is diminished in PCOS iPSC-derived ECs, supporting a central role for altered cell cycle regulation in distinguishing EC states between PCOS and control conditions. The distribution of specific phases of the cell cycle in the iPSC-derived ECs was analyzed by flow cytometry with a BrdU incorporation assay. A significant decrease in the S-phase and arrest in the G1-phase were noted in the PCOS iPSC-derived ECs (**Figures 4A, B**), which reduced the number of proliferating cells compared to the control iPSC-derived ECs. The expression of the cell cycle proteins Cyclin D1 and Cyclin E1 was significantly lower in PCOS iPSC-derived ECs than in control (**Figures 4C, D**), which might be associated with reduced cellular proliferation and cell cycle arrest. Original western blot images have been provided (**Supplementary Figure 4**).

### Impaired DHT-induced cellular proliferation in the PCOS iPSC-derived ECs

**Figure 5:**
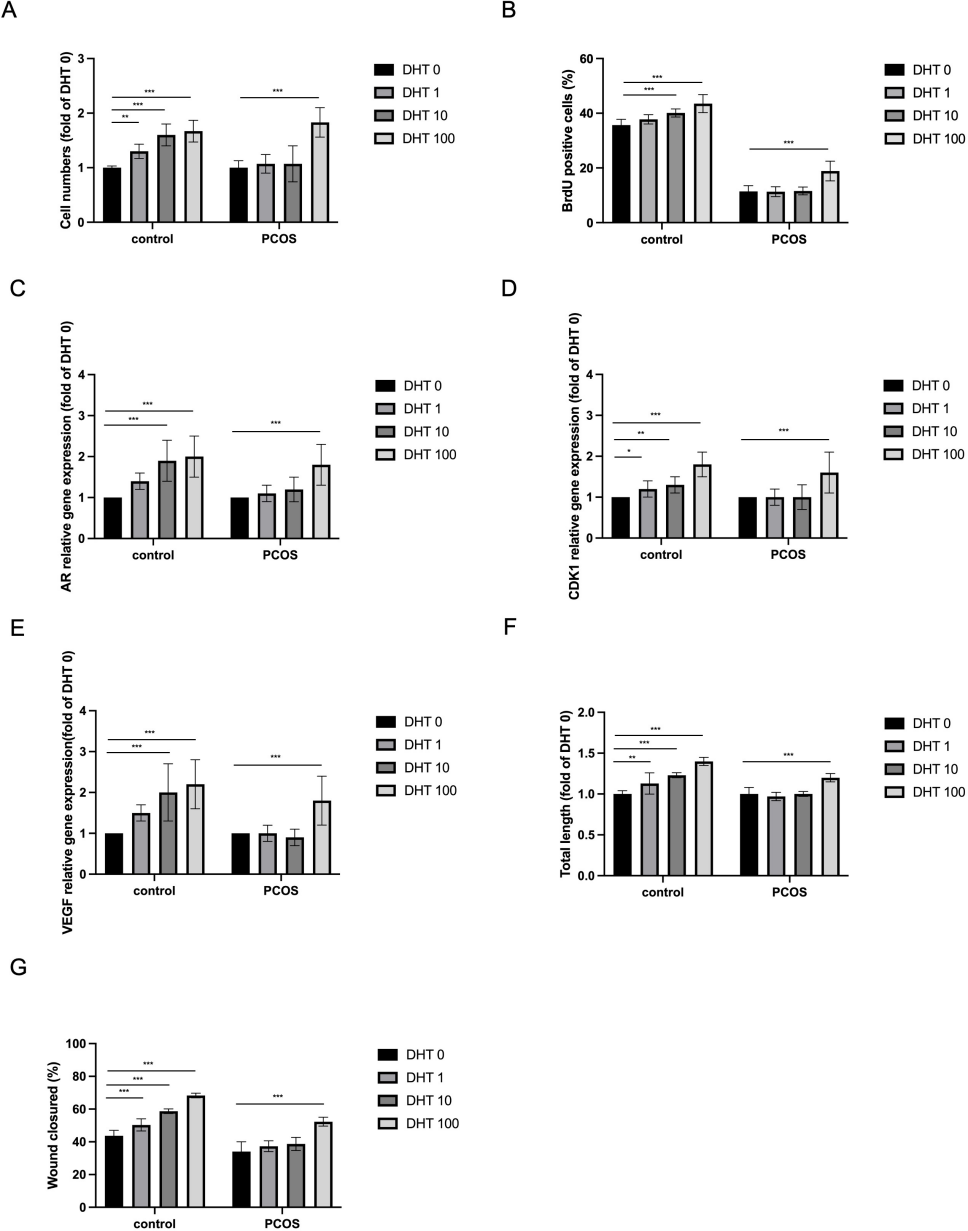
Effects of dihydrotestosterone (DHT) on the growth and function of iPSC-derived ECs. DHT at physiological concentrations (1 and 10 nM) induced proliferation of control iPSC-ECs (N1+N7+N8) but not PCOS iPSC-ECs (P1+P5+P7). **(A, B)** Quantification of cell numbers and BrdU^+^ cells after incubation of iPSC-ECs at different concentrations of DHT (0, 1, 10 or 100 nM) for 24 hr. DHT induced cellular proliferation in control iPSC-derived ECs (N1+N7+N8) at all different doses. Significantly increased cellular proliferation was observed only after the treatment of the highest concentration of DHT (100 nM) in PCOS iPSC-derived ECs (P1+P5+P7). **(C–E)** Relative expression of androgen-induced cellular proliferation genes, androgen receptor (AR), CDK1 and vascular endothelial growth factor (VEGF), was determined in DHT-treated iPSC-derived ECs by Real-Time RT-PCR. DHT significantly increased the expression of AR, CDK1 and VEGF genes in control iPSC-derived ECs (N1+N7+N8) at all different concentrations. In contrast, significantly upregulation of these gene was only observed at 100 nM DHT (100 nM) in PCOS iPSC-derived ECs (P1+P5+P7). **(F)** DHT significantly promoted tube formation at all different concentrations (1, 10 or 100 nM) in control iPSC-derived ECs (N1+N7+N8). In contrast, significantly enhanced tube formation was only observed under the treatment with the highest concentration of DHT (100 nM) in PCOS iPSC-derived ECs (P1+P5+P7). **(G)** The effect of DHT on cell migration was evaluated using a wound healing assay after 24 hr of treatment with varying DHT concentrations (0, 1, 10, and 100 nM). DHT significantly promoted cell migration in control iPSC-derived ECs (N1+N7+N8) at all concentrations, whereas a significant increase in wound closure area was observed in PCOS iPSC-derived ECs (P1+P5+P7) only at 100 nM. Grouped data represented as mean ± SD, control (N1+N7+N8) and PCOS (P1+P5+P7), were used for comparison (n=9 independent experiments). *P<0.05; **P<0.01; ***P<0.001 vs DHT 0 nM.

Given that our scRNA-seq analysis revealed an intrinsic dysregulation of the androgen signaling pathway among the DEGs in PCOS iPSC-derived ECs (**Figure 1C**), we sought to determine whether this transcriptomic signature translates to functional impairments. Viable cell numbers and BrdU-positive cells significantly increased in the control iPSC-derived ECs treated with 1, 10, and 100 nM DHT for 24 hr (**Figures 5A, B**). In contrast, DHT did not promote the proliferation of PCOS iPSC-derived ECs at concentrations of 1 and 10 nM for 24 hr. Significantly increased cellular proliferation was observed only after treatment with the highest concentration of DHT (100 nM) in the PCOS iPSC-derived ECs. The distribution of specific phases of the cell cycle in the iPSC-derived ECs treated with 1, 10, or 100 nM DHT is shown (**Supplementary Figure 5**). The expression of molecules involved in androgen-induced cellular proliferation, including androgen receptor (AR), CDK1, and VEGF was examined in the DHT-treated iPSC-derived ECs by Real-Time RT-PCR (**Figures 5C–E**). The expression of AR, CDK1, and VEGF was significantly increased by treatment with 1, 10, and 100 nM DHT in the control iPSC-derived ECs. However, in the PCOS iPSC-derived ECs, the expression of AR, CDK1, and VEGF did not increase after treatment with DHT at lower doses (1 and 10 nM), but only increased under the highest concentration of DHT (100 nM). These data suggest that DHT-induced cellular proliferation and the expression of associated genes were more sensitive and dose-dependent in the control iPSC-derived ECs and blunted in the PCOS iPSC-derived ECs, while only the highest concentration of DHT upregulated gene expression and stimulated cellular proliferation in the PCOS group.

### Impaired DHT-induced angiogenesis in PCOS iPSC-derived ECs

The modulating effects of androgen on the iPSC-derived ECs in angiogenesis were evaluated using tube formation assay (**Figure 5F**; **Supplementary Figure 6A**) and wound healing assay (**Figure 5G**; **Supplementary Figure 6B**). Significantly enhanced tube formation and cell migration were observed after DHT treatment in a dose-dependent manner in control iPSC-derived ECs. However, DHT was able to induce tube formation and cell migration only at the highest concentrations (100 nM) in PCOS iPSC-derived ECs. These data suggest that DHT-induced angiogenesis was more sensitive in the control iPSC-derived ECs and was blunted in the PCOS iPSC-derived ECs.

## Discussion

In the present study, disease-specific iPSC-derived ECs were successfully established and served as an effective platform for disease modeling to elucidate the mechanisms of EC dysfunction in PCOS. The differentiation efficiency was comparably high between the PCOS and control groups. All the iPSC-derived ECs efficiently expressed endothelial markers and demonstrated endothelial functions. Analysis using scRNA-seq and pathway enrichment revealed functional differences between PCOS and control iPSC-derived ECs, involving cell proliferation, immune responses, metabolic processes, development, cardiovascular functions, apoptosis, cell death, and androgen signaling. Meanwhile, impaired cellular proliferation and decreased expression of proliferation-associated genes were also noted in the PCOS iPSC-derived ECs compared with the control group. Given that the expression of certain endothelial-specific transcription factors in iPSC-derived ECs can be influenced by the original somatic cell source (29), we utilized two iPSC sources, fibroblast-derived (N1 and P1) and PBMC-derived (N7 and P5), to minimize somatic cell source-related variability when comparing scRNA-seq data between control and PCOS iPSC-derived ECs. Our results demonstrated significant differences in cell proliferation between control and PCOS iPSC-derived ECs, regardless of whether they were derived from fibroblasts or PBMCs. This further confirms that the observed differences between control and PCOS iPSC-derived ECs are independent of somatic cell origin. Our data are supported by a previous study showing a decreased number and function of circulating endothelial progenitor cells (EPCs) in non-obese PCOS women compared with age- and BMI-matched controls (31). The number and proliferation ability of EPCs are closely related to neoangiogenesis and vascular repair and are diminished in patients with various cardiovascular risks and morbidities, including obesity, T2DM, HTN, and thromboembolic events (32–35). Our data revealed evidence of endogenous proliferative dysfunction of ECs in the PCOS group, which might indicate an increased risk of unrepaired vascular injury and further cardiovascular risk in women with PCOS.

Although several studies have proposed a positive correlation between androgen excess and signs of EC dysfunction in PCOS, whether and how androgens modulate the growth and function of ECs in women is still unclear. Under physiological conditions, androgen is involved in the proliferation, mobility, adhesion, and regulation of vascular tone, and is beneficial for the growth and function of ECs (12). Androgens induce EC proliferation through the AR/VEGF/cyclin-A-mediated pathway, which could assist in the repair of endothelial injury (36). Nevertheless, there seems to be a dose-dependent and sex-specific impact of androgens on the health of ECs (12). In this study, DHT induced significant cellular proliferation among control iPSC-derived ECs in a dose-dependent manner from 1 to 10 nM, corresponding to physiological serum concentrations in adult women and men (24, 25). This was concordant with previous studies, suggesting the direct beneficial effects of androgens on ECs by enhancing NO production, proliferation, motility, and growth (12). Moreover, real-world longitudinal cohort studies also demonstrated the benefits of androgens for the cardiovascular system in that there was a link between androgen deficiency, HTN, and metabolic syndrome in men and postmenopausal women (37). In our study, blunted responses were noted among PCOS iPSC-derived ECs, as the growth of ECs was not induced by DHT treatment at physiological concentrations and was enhanced only under extreme supraphysiological concentrations. Impaired androgen-induced angiogenesis was also revealed in the PCOS iPSC-derived ECs by functional assays. Taken together, the underlying mechanism of endothelial dysfunction in PCOS is caused by attenuation of the beneficial actions of androgen on ECs rather than direct harm from excess androgen.

In control iPSC-derived ECs, treatment with varying doses of DHT upregulated the expression of AR, VEGF, and CDK1, which was accompanied by enhanced cellular proliferation and angiogenesis. This was concordant with a previous study using primary human aortic ECs, indicating that DHT acts on AR to stimulate EC proliferation through the upregulation of CDK1 and VEGF (36). The authors used AR siRNA, VEGF siRNA, and cyclin-dependent kinase inhibitor to demonstrate that androgen stimulates cell proliferation through the AR/VEGF/CDK1 pathway. In contrast, our study reveals that PCOS iPSC-ECs exhibit a blunted response to DHT at physiological concentration, characterized by diminished responsiveness of the AR/VEGF/CDK1 signaling pathway. These distinct regulatory defects are preserved even after reprogramming and differentiation, providing a molecular basis for the intrinsic angiogenic defects observed in PCOS iPSC-derived cells. Crucially, our scRNA-seq analysis at baseline reveals that the androgen signaling pathway is significantly enriched among the DEGs between PCOS and control ECs. This indicates a fundamental transcriptomic dysregulation of the androgen-responsive machinery in PCOS iPSC-derived cells even prior to exogenous hormonal challenge. While this study primarily focuses on mRNA expression, the significant impairment in tube formation and cell proliferation serves as a functional proxy for the diminished activity of the corresponding proteins. This ‘blunted’ response suggests a fundamental disruption in the signaling cascade where AR typically drives VEGF, cyclin, and CDK1 expression (36). Such dysregulation may result from upstream factors intrinsic to the PCOS genetic background. For instance, AR gene polymorphisms, specifically the length of CAG repeats (38), and other point mutations (39), are known to significantly modulate the transcriptional activity of AR and are associated with an increased risk of PCOS. Additionally, since VEGF-mediated angiogenesis and endothelial repair are critically modulated by androgen/AR signaling pathways, VEGF gene polymorphisms that influence VEGF secretion patterns in PCOS patients could potentially render PCOS iPSC-derived ECs less responsive to DHT (40, 41). Furthermore, epigenetic modifications, such as aberrant DNA methylation of the AR gene promoter and genes within VEGF signaling pathway, may be preserved during the iPSC reprogramming process as ‘epigenetic memory’ (42–44). This preservation could explain the sustained desensitization of the AR/VEGF signaling observed in our model. Other studies have further reported this by reporting differential gene polymorphisms, DNA methylation, and miRNA regulation targeting AR, CDK, and VEGF in women with and without PCOS (39, 45, 46). Future studies utilizing protein-specific inhibitors or CRISPR-mediated epigenetic editing will be instrumental in further dissecting these complex regulatory layers. Clinically, metformin, a widely used drug for PCOS treatment with known benefits for endothelial function and androgen reduction (47), presents a compelling candidate for influencing the AR/VEGF/CDK1 pathway in endothelial cells of PCOS women. Further studies designed to investigate the therapeutic effects of metformin and other relevant pathway regulators on the functional impairments we identified could have significant clinical implications.

Our scRNA-seq analysis reveals an intrinsic reduction of CDK1 transcripts in PCOS iPSC-ECs. The dysregulation of the DHT/AR/VEGF/CDK1 signaling pathway further provides a robust transcriptional basis for this variation. These stand in contrast to the canonical view of CDK1 being constitutively expressed as a stable cell cycle regulator. The pathological state of PCOS might disrupt this homeostatic stability, a view supported by extensive research in other PCOS-affected tissues. For instance, in PCOS granulosa cells (GCs), the stability and expression of CDK1 are actively modulated by the USP25/PI3K/AKT axis to regulate proliferation and apoptosis (48). Similarly, the non-coding RNA circ_0043533 has been identified to facilitate abnormal GC proliferation in PCOS by dysregulating downstream targets, including CDK1 (49). Given that CDK1 is a potent oncogene, its dysregulation carries profound pathogenic relevance in the PCOS landscape. Elevated CDK1 is linked to increased risks of endometrial malignancies in PCOS patients (50), while in endothelial cells, CDK1 expression is critical for vascular health. Silencing CDK1 has been reported to significantly inhibit EC proliferation, migration, and capillary-tube formation (51). By integrating these findings, we clarify that the observed variation in CDK1 is a critical pathological feature of PCOS, serving as a shared molecular denominator for complications ranging from hyperplastic risks to systemic vascular dysfunction.

The iPSC-derived EC model employed in this study offers a powerful platform for investigating the molecular drivers of PCOS-related vascular dysfunction. The validity of this model is substantiated by the robust expression of canonical endothelial markers (e.g., CD31, vWF) and the capacity for angiogenic tube formation, which closely recapitulate the gene expression profiles and functional behaviors of primary endothelial cells. The reliability of our model is underscored by the consistent recapitulation of cell cycle arrest across three independent patient-derived clones. Despite the significant clinical and phenotypic heterogeneity inherent in PCOS, iPSC-derived ECs from different donors in our study converged on a similar pathological defect in proliferation and angiogenic signaling. To ensure experimental reproducibility and minimize phenotypic drift, all assays were strictly conducted using cells between passage 2 and 5, a window where iPSC-derived ECs maintain peak phenotypic stability and endothelial marker expression (52). Traditionally, models of PCOS EC dysfunction have relied on primary ECs (e.g. human umbilical vein endothelial cells (HUVECs) and endothelial progenitor cells (EPCs)) or animal models. However, primary cells often exhibit limited lifespans, significant donor variability, and limited numbers for large studies. While animal models are invaluable for systemic studies, they frequently fail to replicate human-specific vascular responses due to interspecies biological discrepancies (53). In contrast, iPSC-derived ECs offers a renewable, patient-specific source that provides a standardized platform capturing the intrinsic molecular signatures of the donor’s genetic background (54). Although our model has certain limitations, such as a relatively immature phenotype, the lack of interaction with other cell types like immune cells and microenvironment similar to blood vessels, its ability to reliably reproduce the PCOS vascular phenotype across different patients carries significant clinical implications (54). This platform provides a scalable and robust ‘disease-in-a-dish’ system for future personalized drug screening aimed at restoring endothelial health in women with PCOS.

A key concern of this study is the limited number of patient-derived iPSC lines, which reflects the substantial technical complexity, cost, and quality control requirements inherent to iPSC generation and differentiation into stable endothelial cells. Given the marked heterogeneity of PCOS, we deliberately focused on patients with classical, full-blown phenotype A, the most severe and clinically relevant form associated with the highest risk of endothelial dysfunction, to reduce phenotypic variability and enhance mechanistic interpretability. Despite the limited sample size, the observed phenotypes were consistent across independent PCOS iPSC-EC lines. Notably, the use of two to three disease-specific iPSC lines is not uncommon in mechanistic iPSC studies of highly heterogeneous complex disorders, including pulmonary arterial hypertension (55) and bipolar disorder (56), where similar sample sizes have been employed in previous publications due to comparable constraints in differentiation stability and experimental reproducibility. Nevertheless, these findings should be regarded as hypothesis-generating and warrant validation in larger and more diverse cohorts. An important consideration is the potential confounding effect of BMI, as endothelial phenotypes observed in iPSC-derived cells cannot be attributed solely to PCOS. In the present study, PCOS-derived iPSC lines were obtained from women with obesity, whereas control iPSC lines were derived from individuals with normal BMI. However, PCOS is widely regarded as a disorder driven by the interaction between hyperandrogenism and metabolic dysfunction, including obesity; therefore, this study focused on classical phenotype A PCOS, which carries the highest cardiometabolic and endothelial risk. Experimental evidence indicates that obesity can induce PCOS-like features and exacerbate androgen excess (57), suggesting that the observed endothelial abnormalities may reflect amplified androgen signaling under metabolic stress rather than isolated effects of BMI or androgen alone; accordingly, these findings should be considered preliminary and hypothesis-generating and warrant validation in larger, BMI-matched cohorts.

## Conclusion

In conclusion, PCOS-specific iPSC-derived ECs were successfully established in the present study and served as an effective tool for investigating the pathogenesis of EC dysfunction. This is the first study revealing direct evidence of both endogenous and androgen-mediated EC dysfunction in PCOS in a cell-based model. Impaired cellular proliferation and DEGs involved in cell proliferation, immune responses, cardiovascular functions, apoptosis and androgen signaling were observed among PCOS iPSC-derived ECs. Additionally, androgen-induced EC proliferation and angiogenesis were blunted in PCOS through a diminished AR/VEGF/CDK1 signaling pathway, which might hinder VEGF-dependent vascular repair and potentially increase cardiovascular risks. Our results can be applied in further mechanistic research and in the search for possible therapeutic targets in the future.

## Data Availability

The datasets presented in this study can be found in online repositories. The names of the repository/repositories and accession number(s) can be found below: Gene Expression Omnibus (GEO) under accession number GSE246456.
