# Supplementary material for: Pathophysiology of androgen-associated endothelial cell dysfunction in phenotype A polycystic ovarian syndrome revealed by iPSCs modeling
Source: Front Endocrinol (Lausanne). 2026 Feb 12;17:1682793. doi: 10.3389/fendo.2026.1682793 (PMC12935609; doi:10.3389/fendo.2026.1682793)
Supplement: Supplementary file 1 [file DataSheet1.pdf]

Supplementary Data

Supplementary Table 1. The demographic and clinical characteristics of three PCOS patients and control from whom the samples were derived to establish iPSCs.

**PCOS patients**	**P1**	**P5**	**P7**
Age (year)	18	26	25
BMI (kg/m^2)	30.1	32.4	32.6
Weight (kg)	80	81	78.4
Height (cm)	163	158	155
Waist circumference (cm)	101	100.5	97
Hip circumference (cm)	108	109	106.5
Menstrual interval (day)	360	120	360
Polycystic ovarian morphology	+	+	+
Hyperandrogenism			
Alopecia	-	-	+
Acne	-	-	-
Hirsutism	+	-	+
Hormonal profiles			
Total testosterone (ng/ml)	1.2	1.14	0.76
DHEA-S (umol/l)	8.47	6.89	17.82
FSH (mIU/ml)	4.66	5.4	7.72
LH (mIU/ml)	4.67	9.0	10.2
Estradiol (pg/ml)	35.7	67.1	39.3
Progesterone (ng/ml)	0.489	0.7	0.466
Metabolic profiles			
Insulin (uIU/ml)	16.6	15.2	58
Fasting glucose (mg/dl)	89	86	84
75g OGTT 1hr (mg/dl)	176	188	222
75g OGTT 2hr (mg/dl)	121	198	78
HOMA-IR	3.6	3.2	1.2

T-CHO (mg/dl)	207	179	273
LDL-C (mg/dl)	152	122	178
HDL-C (mg/dl)	42	46	72
TG (mg/dl)	152	79	76
AST (U/L)	19	38	15
ALT (U/L)	41	74	12
Systolic blood pressure (mmHg)	130	134	110
Diastolic blood pressure (mmHg)	73	77	74
Heart rate (per minute)	66	89	83

Abbreviations: ALT: alanine aminotransferase, AST: aspartate aminotransferase, BMI: body mass index, DHEA-S: dehydroepiandrosterone sulfate, FSH: follicle-stimulating hormone, HDL-C: high-density lipoprotein-cholesterol, HOMA-IR: homeostasis model assessment-insulin resistance, LDL-C: low-density lipoprotein-cholesterol, LH: luteinizing hormone, OGTT: oral glucose tolerance test, T-CHO: total cholesterol, TG: triglycerides.

**Control**	**N1**	**N7**	**N8**
Age (year)	37	33	28
BMI (kg/m^2)	23	-	22

Supplementary Table 2. The sequences of qPCR probes

VEGF-F	TGCAGATTATGCGGATCAAACC
VEGF-R	TGCATTCACATTTGTTGTGCTCTGTAG
CDK1-F	GGAAACCAGGAAGCCTAGCATC
CDK1-R	GGATGATTCAGTGCCATTTTGCC
AR-F	AGGATGCTCTACTTCGCCCC
AR-R	ACTGGCTGTACATCCGGGAC
SMC4-F	GAGAAAATTCTGGGACCTT
SMC4-R	TCTGAATGTCCTTGTGTTCA
TOP2A-F	GCCAGAATCTGTTCGCTTCAAC
TOP2A-R	AGGAAACTGAGTGCCGGCTT
MKI67-F	TCCTTTGGTGGGCACCTAAGACCTG
MKI67-R	TGATGGTTGAGGTCGTTCCTTGATG
DEK-F	AGGAGGAAGAGGACGAGGAC
DEK-R	GGAAAGCCACTGAACTGACC
eEF1a1-F	GCCAGAACACAGGTGTCGTGAAAAC
eEF1a1-R	CAGATGGCCAGTAGTGGTGGACTTG

Supplementary Table 3. Parameters of scRNA-seq data.

**Parameter**	**N1**	**N7**	**P1**	**P5**
Number of Cells	9,713	12,430	12,165	13,440
Number of Reads	430,145,821	377,059,570	373,381,338	364,401,089
Valid barcodes (%)	97.40%	97.50%	97.60%	97.40%
Mean Reads/ Cell	44,286	30,335	30,693	27,113
Median Genes/ Cell	4,500	3,700	3,625	3,268
Median UMI counts/ Cell	17,876	11,648	11,583	10,406

Supplementary Figure 1

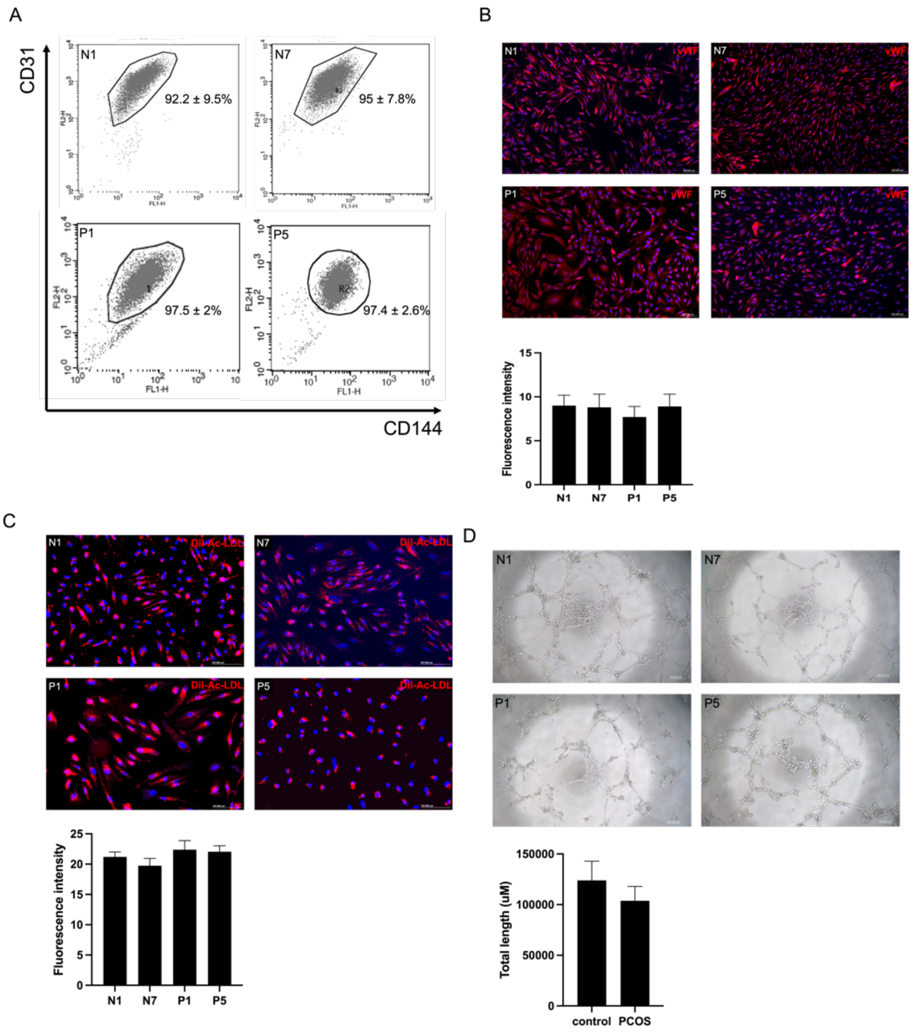

Legend: **Differentiation of endothelial cells (ECs) from two control (N1 and N7) and two PCOS (P1 and P5) induced pluripotent stem cells (iPSCs).** (**A**) EC surface markers, CD31 and CD144, were analyzed at passage 2 of control and PCOS iPSC-ECs by flow cytometry. ECs were gated as CD31$^+$CD144$^+$ double positive cells. Percentages of gated populations were indicated. Up to 90% of iPSC-derived ECs were generated from both control and PCOS iPSCs. (**B**) Immunostaining for von Willebrand Factor (vWF) (red) and Hoechst 33342 (nuclei, blue) was evaluated in control and PCOS iPSC-derived ECs. There was no significant difference in the mean immunofluorescence intensity of vWF between PCOS and control iPSC-derived ECs. (**C**) Functional activities of iPSC-derived ECs were examined by the abilities of Dil-

Ac-LDL up-taking and tube forming on Matrigel. Representative fluorescent images of incorporated Dil-Ac-LDL (red) and Hoechst 33342 (nuclei, blue) in control and PCOS iPSC-derived ECs. Quantification of mean fluorescence intensity of Dil-Ac-LDL indicated absence of significant difference in the PCOS and control iPSC-derived ECs. (**D**) Tube formation was observed on Matrigel. Total tubal length was calculating with the Angiogenesis Analyzer using Fiji plug in for Mac OS X version of ImageJ. The N1 iPSC-derived ECs showed greater ability of forming tube than others. No significant difference in total tube length was observed between control (N1 and N7) and PCOS group (P1 and P5). Data are represented as mean ± SD (n=3 independent experiments). Grouped data, control (N1+N7) and PCOS (P1+P5), were used for comparison (n=6 independent experiments). The t-test was used for comparison between 2 groups. The one-way ANOVA was used for comparison between four iPSC-ECs. Scale bar as indicated.

Supplementary Figure 2

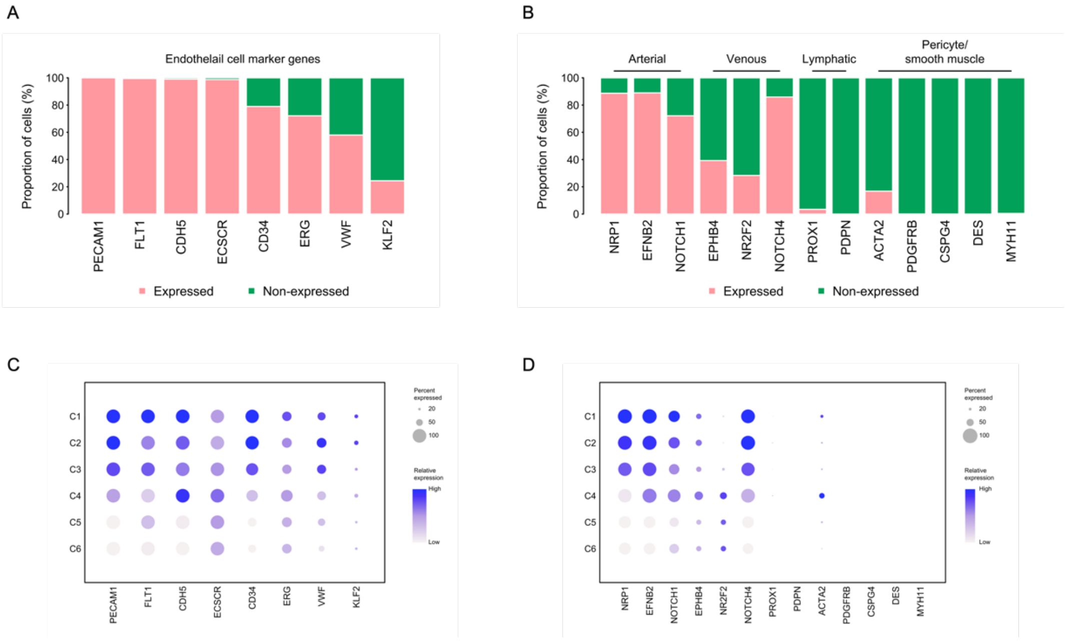

Legend: **Characterization of EC identity and lineage commitment.** (A and B) Bar plots show the proportion of cell expressing each canonical endothelial (A) and vascular subtype marker genes (B). Expression was defined as detectable transcript levels (read count > 1) in individual cells. (C and D) Dot plots showing the distribution of canonical endothelial (C) and vascular subtype marker genes (D) expression across cell clusters. Dot size indicates the proportion of expression cells and color intensity reflects relative expression levels, with darker colors indicating higher expression and lighter colors indicating lower expression. Canonical EC markers include platelet and endothelial cell adhesion molecule 1 (PECAM1), fam related receptor tyrosine kinase 1 (FLT1), cadherin 5 (CDH5), endothelial cell surface expressed chemotaxis and apoptosis regulator (ECSCR), CD34, ETS transcription factor (ERG), and von Willebrand factor

(VWF). Lineage-specific commitment was assessed using markers for arterial (neuropilin 1 (NRP1), ephrin B2 (EFNB2), and notch receptor 1 (NOTCH1)), venous (EPH receptor B4 (EPHB4), nuclear receptor subfamily 2 group F member 2 (NR2F2), and notch receptor 4 (NOTCH4)), and lymphatic (prospero homeobox 1 (PROX1) and podoplanin (PDPN)) ECs. The expression of pericyte markers (platelet-derived growth factor receptor beta (PDGFRB), chondroitin sulfate proteoglycan 4 (CSPG4)) and smooth muscle cell markers (actin alpha 2 (ACTA2), myosin heavy chain 11 (MYH11), desmin (DES)) were also assessed.

Supplementary Figure 3

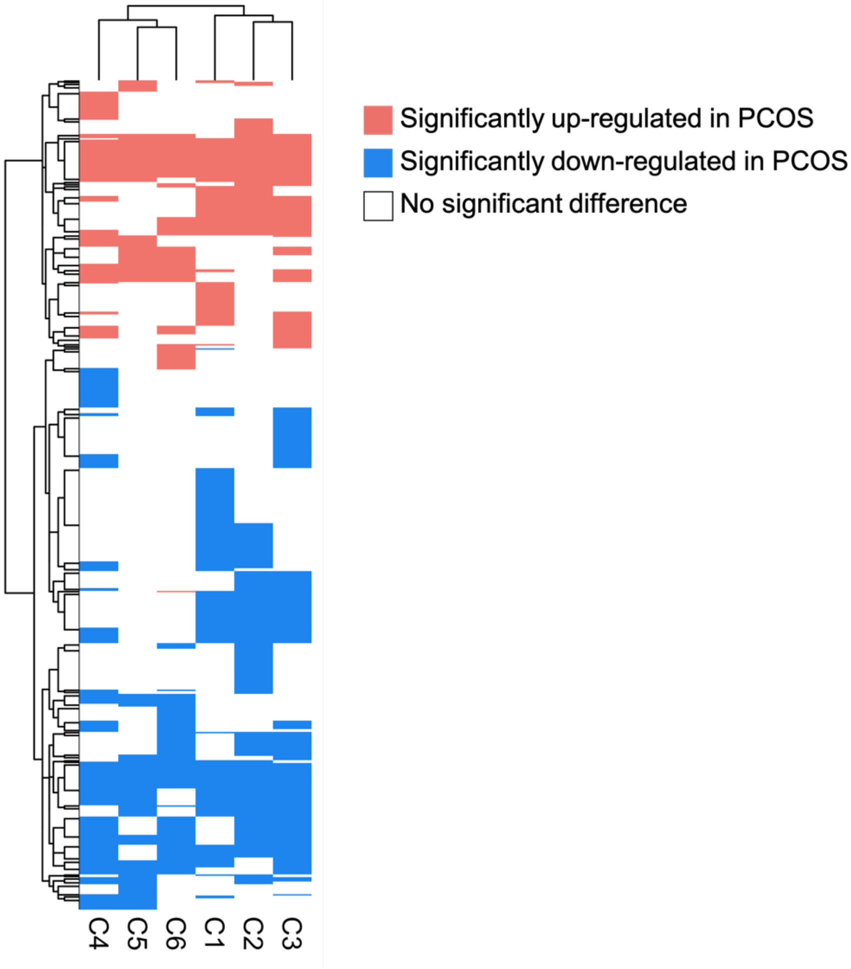

Legend: **Comparative transcriptomic analysis of PCOS and control iPSC-derived ECs across cell clusters.** Heatmap shows hierarchical clustering of genes exhibiting differential expression between PCOS and control iPSC-derived ECs across six cell clusters (C1-C6). Red and blue indicated genes significantly upregulated or downregulated in PCOS iPSC-derived ECs, respectively, while white indicates no significant change.

Supplementary Figure 4

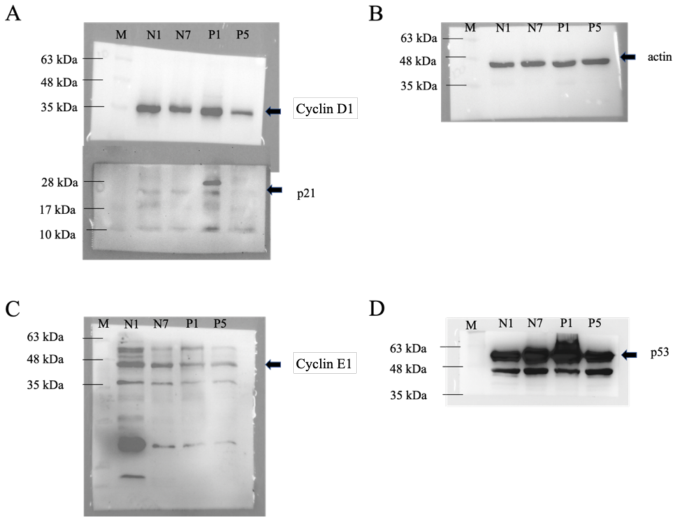

Legend: **Uncropped Western blots.** In our study, single-group samples were subdivided into two aliquots and co-loaded onto a single polyacrylamide gel. (A) The left section of the membrane was excised and subjected to analysis for Cyclin D1 (upper section) and p21 (lower section) with varying exposures times. (B) Following membrane stripping, the upper portion was used for actin analysis. (C) The right segment of the membrane was utilized for Cyclin E1 analysis. (D) Subsequently, after membrane stripping, this segment was further cropped and employed for p53 analysis.

Supplementary Figure 5

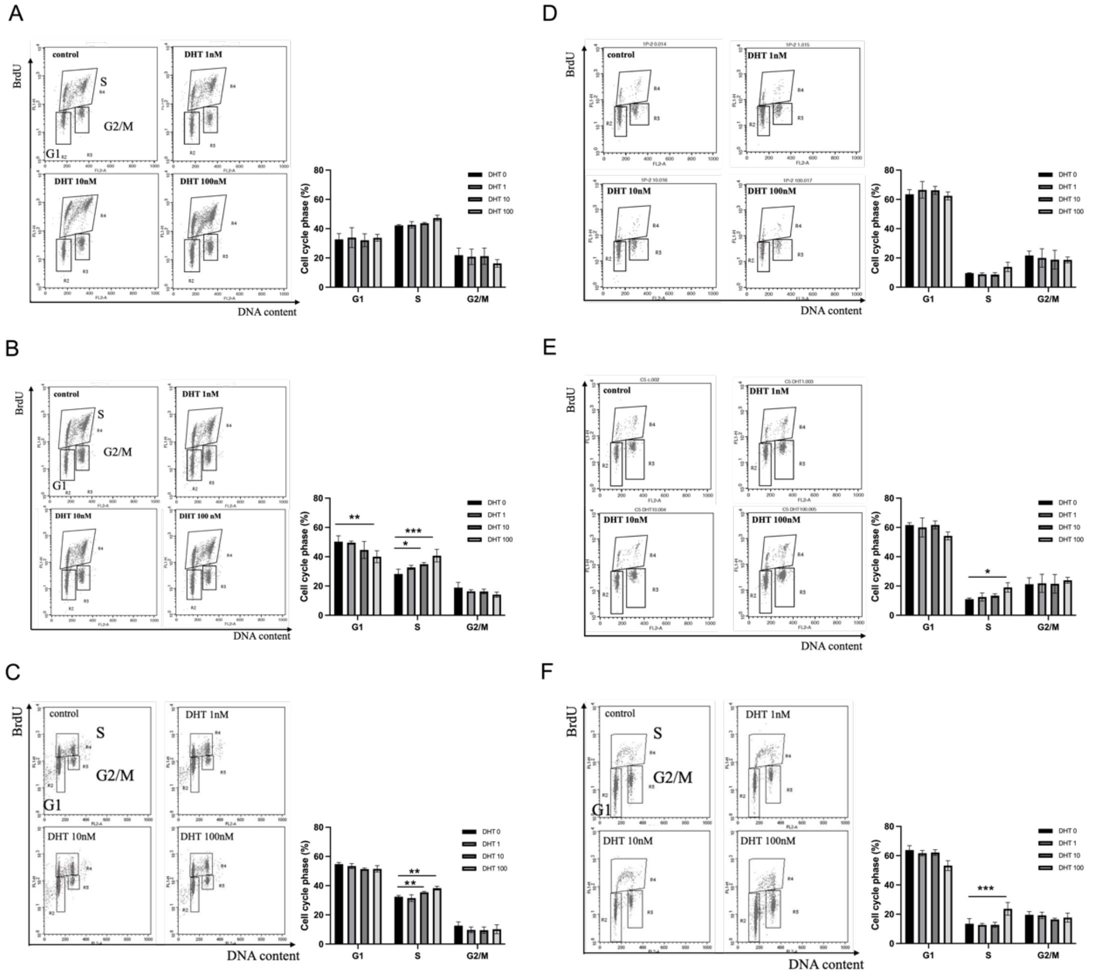

Legend: The distribution of specific phases of the cell cycle in the iPSC-derived ECs treated with 1, 10, or 100 nM dihydrotestosterone (DHT) was analyzed using 5-bromo-2’-deoxyuridine (BrdU) incorporation assay. DHT stimulation does not influence cell cycle phases in iPSC-ECs. Cell cycle distribution and the proportion of cell cycle phases in (A) N1, (B) N7, (C) N8, (D) P1, (E) P5, (F) P7, and results are expressed as mean ± SD in three independent experiments. The separated distribution within S-phase likely reflects the intrinsic replication kinetics and high adhesion profile characteristic of iPSC-derived ECs. All cells in gated region were identified by dual criteria: positive BrdU incorporation and intermediate DNA content between 2n and 4n. The two-way ANOVA followed by Dunnett’s test was used to evaluate the impact of various DHT

concentrations on the cell cycle phases of each individual cell line, with comparisons made solely against the untreated control (0 nM) for each respective phase. $^*P<0.05$; $^{**}P<0.01$; $^{***}P<0.001$ indicate statistical significance.

Supplementary Figure 6

A

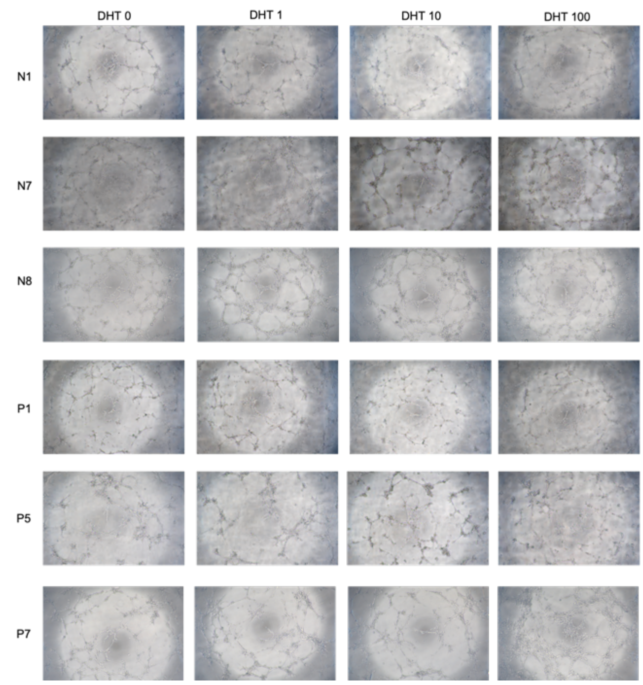

B

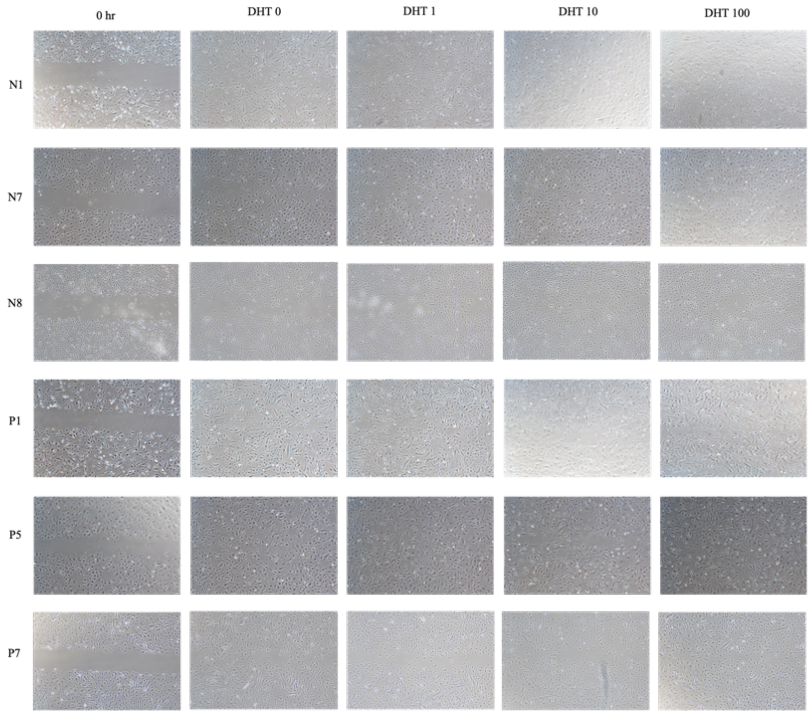

Legend: Functional assays of dihydrotestosterone (DHT)-treated iPSC-ECs. (**A**) DHT significantly promoted tube formation at all different concentrations (1, 10 or 100 nM)

in control iPSC-ECs. Images of tube forming on Matrigel were taken at time 6 hr under x 40 magnification with an inverted microscope. Total length of tubing was calculating with Angiogenesis Analyzer using Fiju plug in for the Mac OS X version of ImageJ. (B) Whether DHT affects cell migration was evaluated using wound healing assay at different concentrations of DHT treatment (0, 1, 10 and 100 nM) for 24 hrs. Images of scratched area were taken at time 0 and time 24 hr with x 40 magnification by using an inverted microscope. Wound closure area was analyzed using Fiji for the Mac OS X version of ImageJ.
